# Supplementary material for: Single-nucleus RNA-sequencing of autosomal dominant Alzheimer disease and risk variant carriers
Source: Nat Commun. 2023 Apr 21;14:2314. doi: 10.1038/s41467-023-37437-5 (PMC10121712; doi:10.1038/s41467-023-37437-5)
Supplement: Supplementary file 1 — Supplementary Information [file 41467_2023_37437_MOESM1_ESM.docx]

*Single-nucleus RNA-sequencing of autosomal dominant Alzheimer disease and risk variant carriers*

Supplementary Information

Logan Brase^1,2,3^, Shih-Feng You^1,2,3^, Ricardo D’Oliveira Albanus^1,2,3^, Jorge L. Del-Aguila^4^, Yaoyi Dai^5^, Brenna C Novotny^1,2,3^, Carolina Soriano-Tarraga^1,2,3^, Taitea Dykstra^6,7^, Maria Victoria Fernandez^1,2,3^, John P Budde^1,2,3^, Kristy Bergmann^1,2,3^, John C Morris^2,8,9^, Randall J Bateman^2,8,9^, Richard J Perrin^2,6,8,9^, Eric McDade^1^, Chengjie Xiong^8,10^, Alison Goate^10,11^, Martin Farlow^10,12^, Dominantly Inherited Alzheimer Network (DIAN), Greg T Sutherland^13^, Jonathan Kipnis^6,7^, Celeste M Karch^1,2,3,*^, Bruno A Benitez^14,*^ and Oscar Harari^1,2,3,*,#^

Affiliations:

1. Department of Psychiatry, Washington University School of Medicine in St. Louis, St. Louis, MO, USA
2. Hope Center for Neurological Disorders, Washington University School of Medicine in St. Louis, St. Louis, MO, USA
3. NeuroGenomics and Informatics, Department of Psychiatry, Washington University School of Medicine in St. Louis, MO, USA
4. Merck & Co., Inc., Boston, MA, USA
5. Baylor College of Medicine, Houston, TX, USA
6. Department of Pathology and Immunology, Washington University School of Medicine in St. Louis, St. Louis, MO, USA
7. Center for Brain Immunology and Glia (BIG), Washington University School of Medicine in St. Louis, St. Louis, MO, USA
8. Knight Alzheimer Disease Research Center, Washington University School of Medicine in St. Louis, St. Louis, MO, USA
9. Department of Neurology, Washington University School of Medicine in St. Louis, St. Louis, MO, USA
10. Division of Biostatistics, Washington University School of Medicine in St. Louis, St. Louis, MO, USA
11. Ronald M. Loeb Center for Alzheimer’s Disease, Department of Genetics and Genomic Sciences, Icahn School of Medicine at Mount Sinai, New York, NY, USA
12. Department of Neurology, Indiana University School of Medicine, Indianapolis, IN, USA
13. School of Medical Sciences and Charles Perkins Centre, Faculty of Medicine and Health, The University of Sydney, Sydney, NSW, Australia
14. Department of Neurology, Beth Israel Deaconess Medical Center, Harvard Medical School, Boston, MA, USA

* These authors contributed equally as co-senior authors

^#^ To whom correspondence should be addressed

**Supplementary Information**

# Contents

Supplementary Results

The inclusion of genetically related individuals did not skew linear regression results2

TREM2 p.R136W clusters with ADAD nuclei2

Coexpression of APOE and MHC-I highlights inhibitory neuron vulnerability to neurodegeneration2

Supplementary Methods

Mic-reduced expression of resting and activated markers3

Supplementary References3

Supplementary Figures

Supplementary Figure 1 – Batch and sample entropy distributions4

Supplementary Figure 2 – Cell type UMAPs by sample5

Supplementary Figure 3 – Full gene estimate heatmaps for sAD, TREM2, and ADAD samples6

Supplementary Figure 4 – ADAD samples have larger effect sizes in general than sAD samples7

Supplementary Figure 5 – DEG set overlaps by genetic status and cell type8

Supplementary Figure 6 – Full cell state GO heatmap9

Supplementary Figure 7 – Cell states enriched with ADAD samples10

Supplementary Figure 8 – Microglia expression of activated and resting marker genes for all cell states11

Supplementary Figure 9 – TREM2 associated clusters replicated in ROSMAP cohort11

Supplementary Figure 10 – ROSMAP microglia cell state signature scores12

Supplementary Figure 11 – ROSMAP oligodendrocyte cell state signature scores13

Supplementary Figure 12 – MS4A cell state proportions by cell type14

Supplementary Figure 13 – Expression Patterns of GWAS Loci14

Supplementary Figure 14 – Replication of prioritized GWAS gene expression patterns in UCI data15

Supplementary Figure 15 – Barcode inflection plots used in quality control filtering15

Supplementary Figure 16 – QC metrics by sample16

Supplementary Figure 17 – QC metrics by cell state16

Supplementary Figure 18 – Neuron cortical layers17

Supplementary Figure 19 – Comparing effects and p-values after removing genetically related individuals18

Supplementary Figure 20 – TREM2 p.R136W clusters with ADAD samples19

Supplementary Figure 21 – Neuronal APOE and MHC1 expression20

Dominantly Inherited Alzheimer Network (DIAN) Author List21

# Supplementary Results

**The inclusion of genetically related individuals did not skew linear regression results**

Three pairs of participants in the discovery cohort are genetically related. Using linear regression, we confirmed that including all individuals instead of one from each pair does not skew our regression analyses. The estimates between the retained and removed models were highly concordant (slope range 0.97 – 1.028; Supplementary Figure 19; Supplementary Dataset 26). The p-values were also highly concordant, with the oligodendrocyte analyses showing more significant p-values when all samples were retained (slope range 0.905 – 1.062). Overall, negligible differences were seen, so all samples were included in the official analyses to maximize power.

**TREM2 p.R136W clusters with ADAD nuclei**

An inspection of the European-descendent *TREM2* p.R136W variant^1^ carrier revealed a divergent signature compared to other *TREM2* carriers. Cellular studies showed that *TREM2* p.R136W severely altered both cell surface and overall TREM2 expression^2^. Three *TREM2* variant carriers were Early-Onset AD (EOAD), but only the p.R136W carrier had a Braak NFT stage of VI (6). In each cell type, this individual's nuclei tended to cluster with the ADAD nuclei (Supplementary Fig 18), suggesting that this mutation could alter expression networks more similarly to *PSEN1* or *APP* mutations than to other *TREM2* variants. Due to the rarity of this variant, only a single brain was analyzed; thus, additional replication is required to validate these observations.­­

**Coexpression of *APOE* and *MHC-I* highlights inhibitory neuron vulnerability to neurodegeneration**

We explored the role of *APOE* expression in neurons. As was previously described^3^, we found a moderate proportion of *APOE*-high (See Methods) expression neurons in controls (20%) with significantly higher proportions in presymptomatic brains (69%, P=6·52×10^-4^) and considerably lower proportions in both sAD (5·7%, P=2·91×10^-2^) and ADAD (3·4%, P=2·20×10^-2^) brains (Supplementary Figure 20a,b). We also observed a correlation between *APOE* and *MHC-I* within specific populations of neurons (Supplementary Figure 20c). Only inhibitory neurons (IN.0 and IN.2) showed significant positive correlations in almost all sub-groups (ADAD, sAD, presymptomatic, and controls), but all inhibitory and excitatory cell states had substantial correlations in ADAD participants (Supplementary Figure 20c). An increased expression of *APOE* and *MHC-I* within a neuron seems to tag it for removal^3^, which agrees with the sharp increase in *APOE-*high neurons before clinical manifestations (presymptomatic) of AD followed by a sharp decrease after (sAD and ADAD). The correlation in IN.0 and IN.2 across sub-groups supports the premise that these inhibitory neurons are the first to succumb to neurodegeneration^4^ and that as pathology progresses, excitatory neurons are also targeted, as seen in the ADAD participants. *APOE*-high nuclei in IN.0 and IN.2 had upregulated genes in 'cellular response to cytokine stimulus' (Adj.P=3·24×10^-10^), suggesting cytokines could trigger the increased *APOE* and *MHC-I* expression in these cells^5^ (Supplementary Dataset 29, Supplementary Dataset 30a). Dysregulated genes were involved in circadian entrainment, cAMP signaling, and NMDA receptor activity as previously reported and highlight sleep and circadian rhythm disruption in AD^6^ (Supplementary Dataset 30a,b).

# Supplementary Methods

**Mic-reduced expression of resting and activated markers**

We used partial residuals from linear mixed models to depict the expression of resting (*TMEM119*, *P2RY13*, *CX3CR1*, *BIN1*, *MED12L*, and *SELPLG*) and activated (*ABCA1*, *RELB*, *GPNMB*, *CD68*, *C5AR1*, *TNFAIP3*, and *CD83*) microglia markers by cell state corrected for sex and age of death. For example, ‘cellState’ is the term of interest in the following model:

(1) count ~ *b*_1_cellState + *b*_2_sex + *b*_3_AOD + *b*_4_(1|subj) + *e*

Each cell state is compared to all other cell states merged as one group. Using the values from fitting the model above, the partial residual or corrected expression (count_c_) is:

(2) count_c_ = *b*_1_cellState + *e*

The partial residual can frequently be negative, so for easier interpretation, we subtracted the minimum corrected expression value and added 0.1 before plotting:

(3) expression = count_c_ − min(count_c_) + 0.1.

# Supplementary References

1 Jin, S. C. *et al.* Coding variants in TREM2 increase risk for Alzheimer's disease. *Hum Mol Genet* **23**, 5838-5846 (2014). <https://doi.org:10.1093/hmg/ddu277>

2 Sirkis, D. W. *et al.* Rare TREM2 variants associated with Alzheimer's disease display reduced cell surface expression. *Acta Neuropathol Commun* **4**, 98 (2016). <https://doi.org:10.1186/s40478-016-0367-7>

3 Zalocusky, K. A. *et al.* Neuronal ApoE upregulates MHC-I expression to drive selective neurodegeneration in Alzheimer's disease. *Nat Neurosci* **24**, 786-798 (2021). <https://doi.org:10.1038/s41593-021-00851-3>

4 Shimojo, M. *et al.* Selective Disruption of Inhibitory Synapses Leading to Neuronal Hyperexcitability at an Early Stage of Tau Pathogenesis in a Mouse Model. *Journal of Neuroscience* **40**, 3491-3501 (2020). <https://doi.org:10.1523/Jneurosci.2880-19.2020>

5 Zhang, H. L., Wu, L. M. & Wu, J. Cross-Talk between Apolipoprotein E and Cytokines. *Mediat Inflamm* **2011** (2011). <https://doi.org:10.1155/2011/949072>

6 Phan, T. X. & Malkani, R. G. Sleep and circadian rhythm disruption and stress intersect in Alzheimer's disease. *Neurobiol Stress* **10** (2019). <https://doi.org:10.1016/j.ynstr.2018.10.001>

# Supplementary Figures

Clicking on the figures will bring you to a full-resolution image.

[
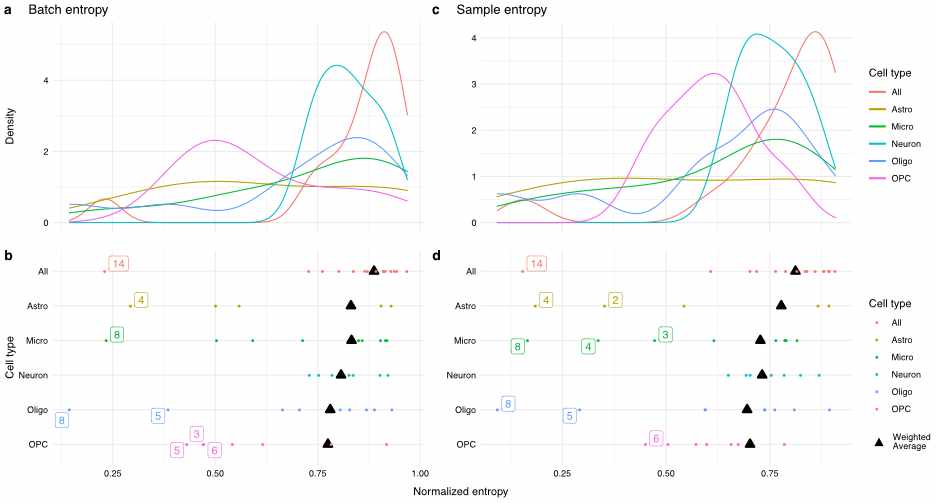
](https://wustl.box.com/s/x3rv0rw4kedopy6zzqutafnqny41l99y)

Supplementary Figure 1 Batch and sample entropy distributions.

a,b) Batch entropy for each cell state. c,d) Sample entropy for each cell state. a,c) Entropy density plots of cell states for each cell type. b,d) Line plots depicting each cell state's normalized entropy. (Δ) indicates the weighted sum or overall entropy for each cell type. 'all' refers to all cell types together, as depicted in Figure 1b. Source data are provided as a Source Data file.

[
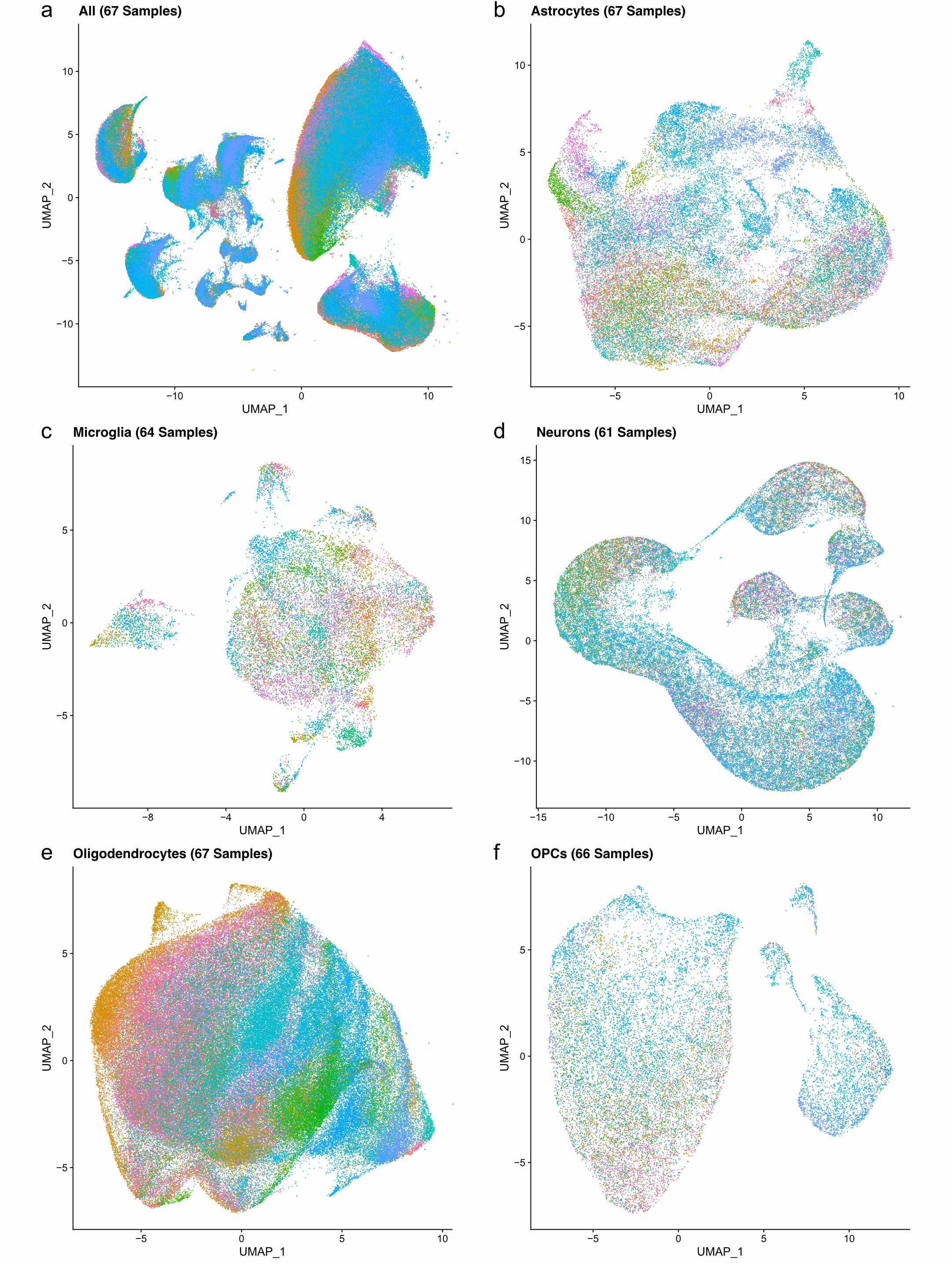
](https://wustl.box.com/s/5wt02sqtdd65v5itjuc1n0dhrl6yzsx5)

Supplementary Figure 2 Cell type UMAPs by sample.
UMAPs for all nuclei and by cell type colored by sample. The number of samples is variable for each plot, as depicted in the plot titles.

[](https://wustl.box.com/s/a9t4ysau2adt33umg6exf56ulgke3k8f)

Supplementary Figure 3 Full gene estimate heatmaps for sAD, TREM2, and ADAD samples.

Full heatmaps of gene estimates from the linear regression models emphasize the divergent and congruent expression patterns across genetic groups (related to Fig. 2b). The largest 500 estimates were selected per cell type. "Modules" were manually created based on expression patterns and dendrogram groupings sporadic ("sAD sig."), TREM2 carriers("TREM2 sig."), and ADAD ("ADAD sig.") depict the significance status of each gene for that group. 'BH' = Benjamini-Hochberg p < 0.05. ‘Nominal’ = p < 0.05. 'NS' = not significant. Source data are provided as a Source Data file.

[
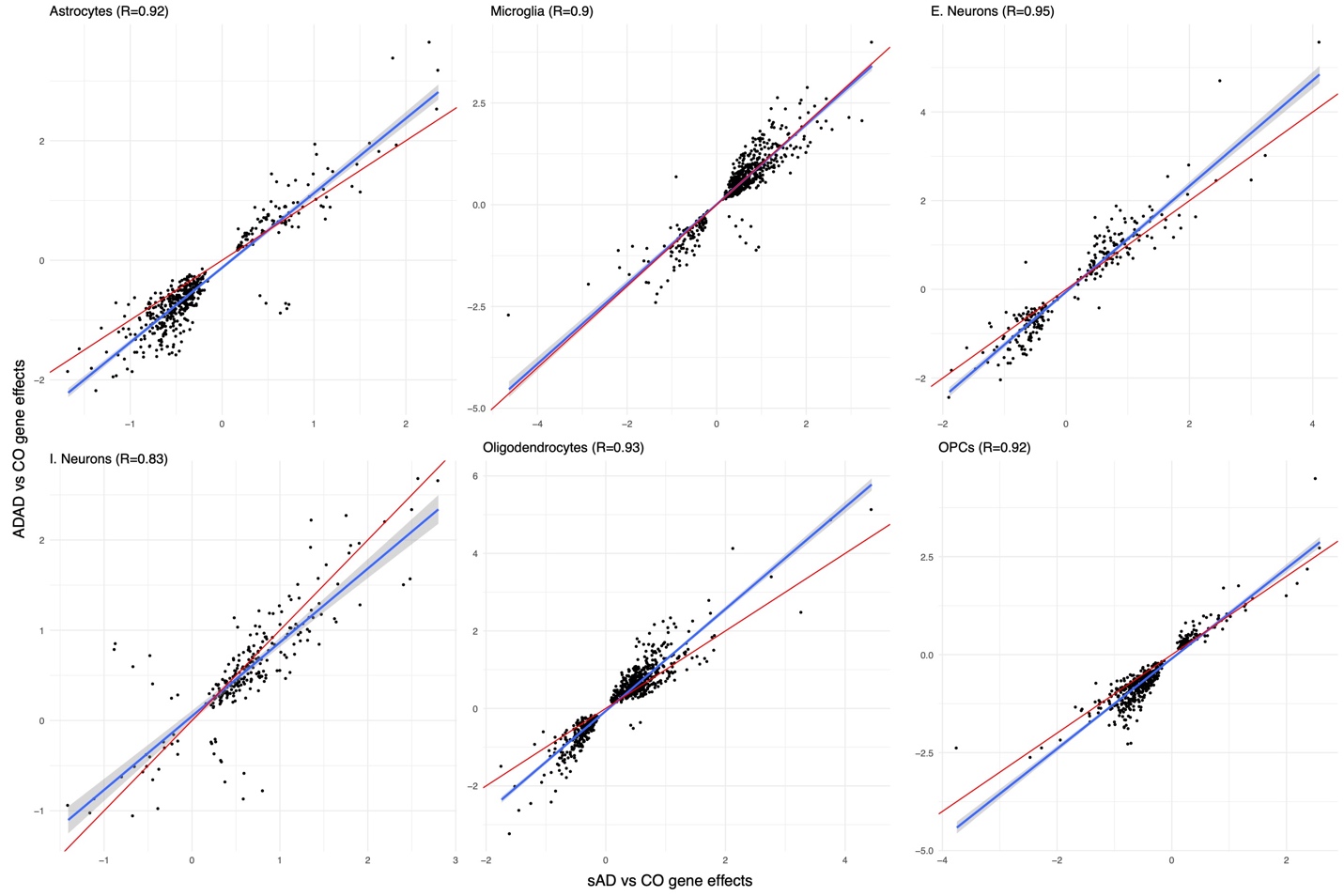
](https://wustl.box.com/s/gyblua7dybwh9g5kzw8f5ge8mzwuljen)

Supplementary Figure 4 ADAD samples generally have larger effect sizes than sAD samples.

Scatterplots comparing the DEG effects of sAD compared to controls and ADAD compared to controls for each cell type. The blue line represents the line of best fit. The red line depicts a perfect 1:1 correlation. When the blue line is rotated counterclockwise compared to the red line, it indicates the ADAD samples have larger effect sizes. The Pearson correlation coefficient (R) is shown for each cell type. Source data are provided as a Source Data file.

[
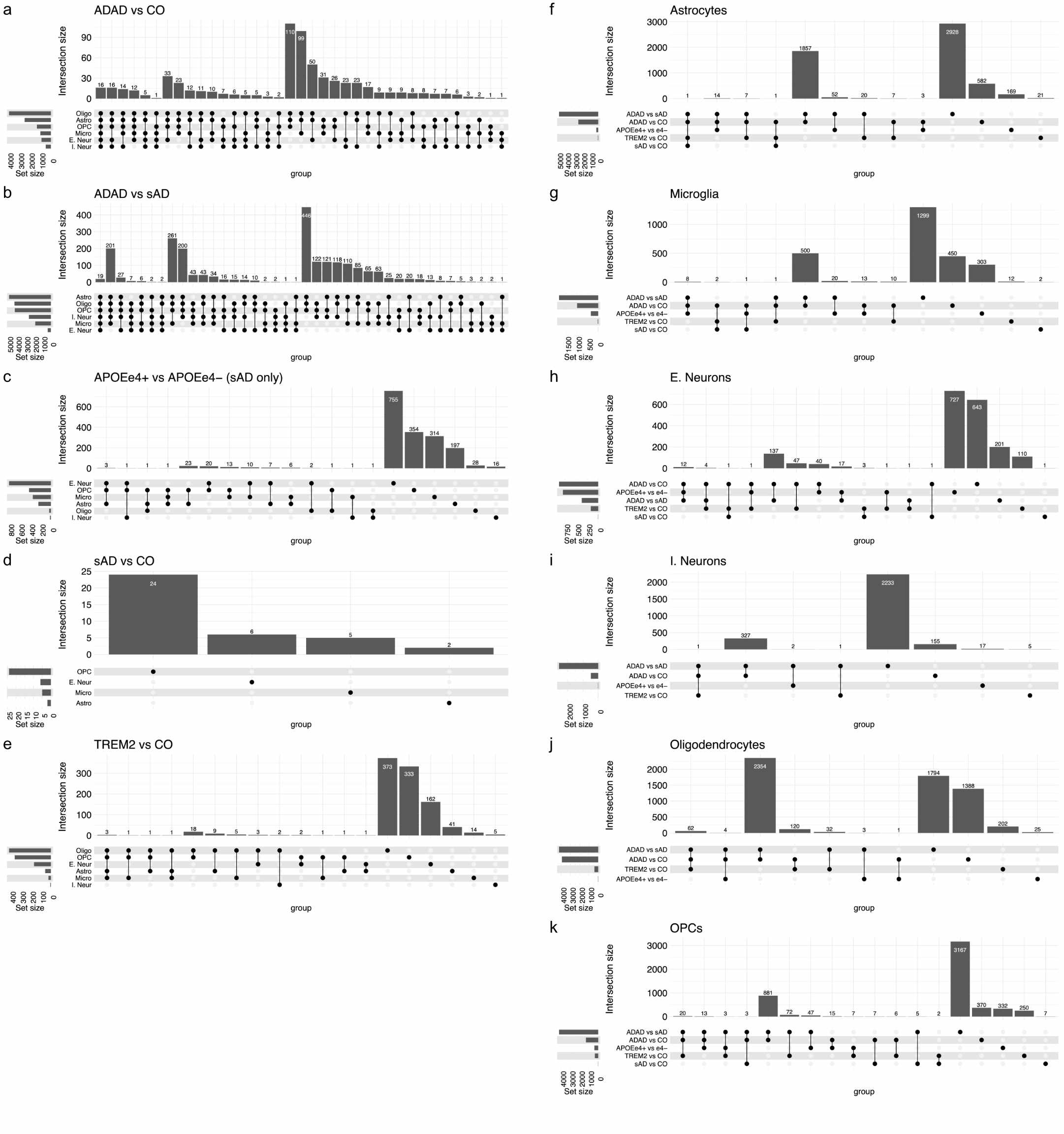
](https://wustl.box.com/s/ymre666tj1cgh7hg5xrzay5wxuw5pnzt)

Supplementary Figure 5 DEG set overlaps by genetic status and cell type.

a-e) Upset plots showing the intersection between cell state DEG sets within genetic statuses. f-k) Upset plots showing the intersection between genetic status DEG sets within cell types. Source data are provided as a Source Data file.

[
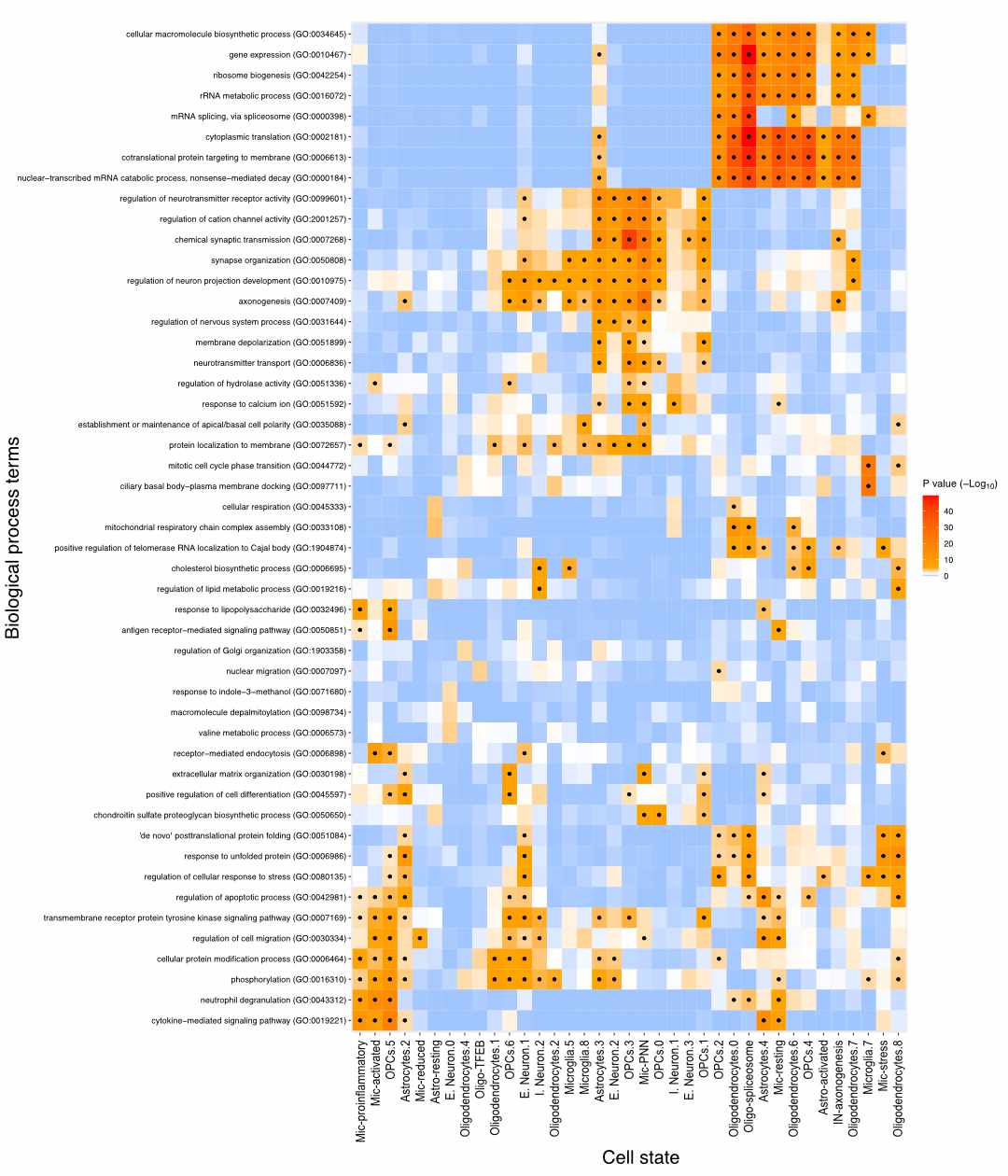
](https://wustl.box.com/s/gsideq0du3e5t634eq0dq3yd2cvfkqph)

Supplementary Figure 6 Full cell state GO heatmap.

A heatmap showing the nominal p-values for each GO term by cell state. This full heatmap includes the terms manually determined to add little information (redundant) even after the systematic summarization and were removed from the Fig. 3g representation. (**⋅**) indicates Benjamini-Hochberg p < 0.05. Source data are provided as a Source Data file.

[
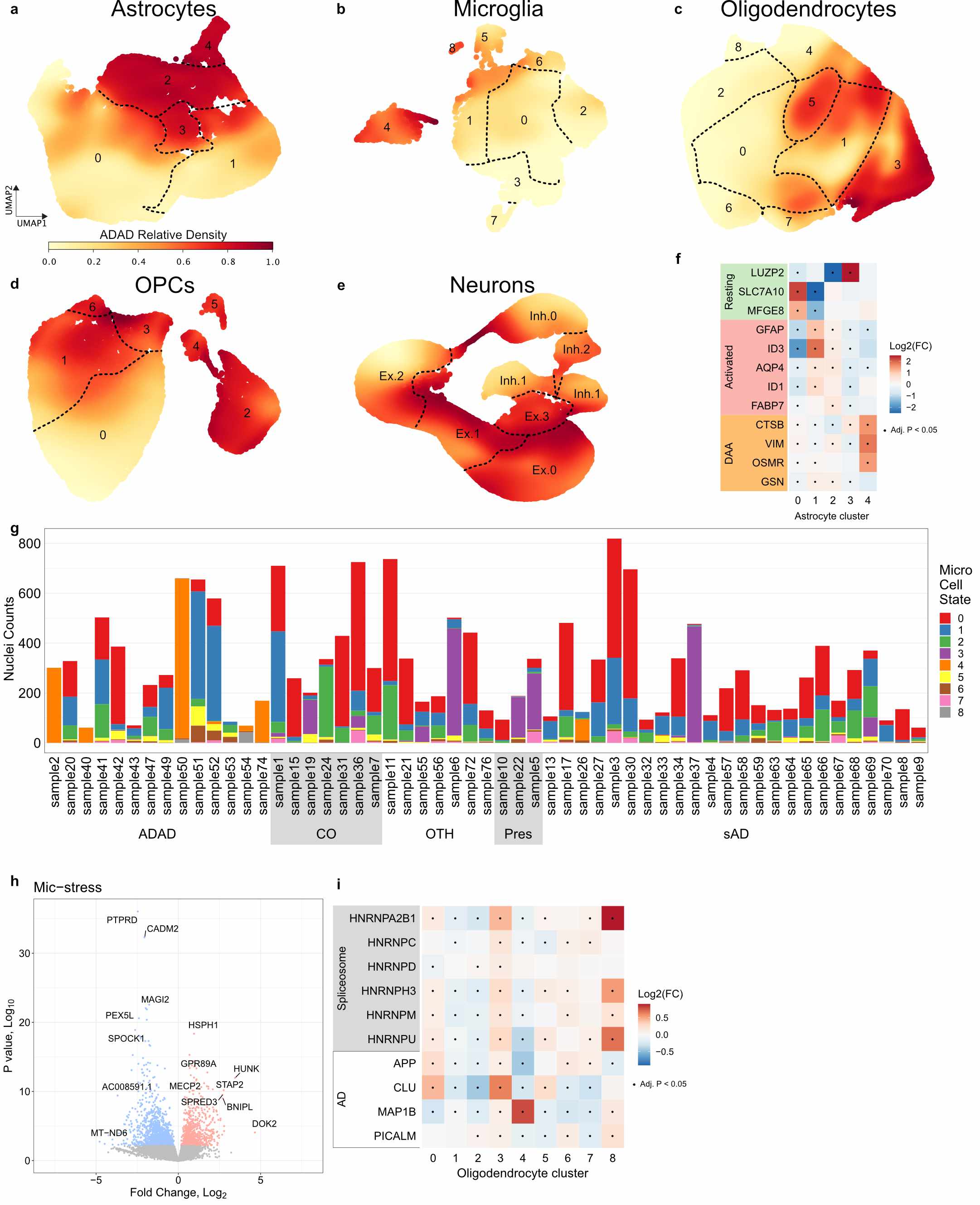
](https://wustl.box.com/s/h8zh1o6alxpsstumzy5rxf42mgxz1xmw)

Supplementary Figure 7 Cell states enriched within ADAD samples.

a-e) Enrichment plots highlighting the density of ADAD nuclei within the UMAP space for each cell type. f) A heatmap showing the log2 fold change in astrocyte resting (LUZP2, SLC7A10, and MFGE8), activated (GFAP, ID3, AQP4, ID1, and FABP7), and DAA (CTSB, VIM, OSMR, and GSN) marker gene expression by cell state. g) Microglia cell state proportions by individual sample. h) Volcano plot of DEGs identified between Mic-stress (Mic.4) and all other microglia cell states. Red points indicate increased expression in Mic-stress. i) A heatmap showing the log2 fold change in splicing-related gene expression for each cell state compared to all other cell states. HNRNP family of genes is directly related to spliceosomes, and APP, CLU, MAP1B, and PICALM are AD genes known to be influenced by this family of genes. Source data are provided as a Source Data file.

[
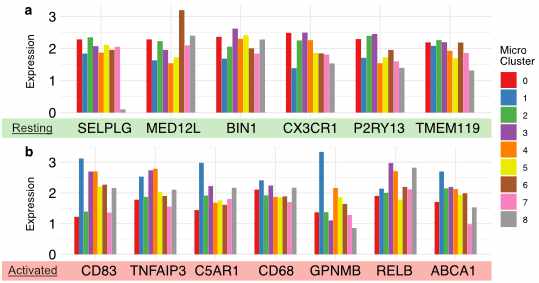
](https://wustl.box.com/s/lha2xn2t7tq9ld2cmwk6ayvhimvoyfh4)

Supplementary Figure 8 Microglia expression of activated and resting marker genes for all cell states.
Barplots showing the expression of (a) resting and (b) activated microglial marker genes for each microglial cell state. Expression was corrected for age of death and sex using partial residuals from linear mixed effects regression. Source data are provided as a Source Data file.

[
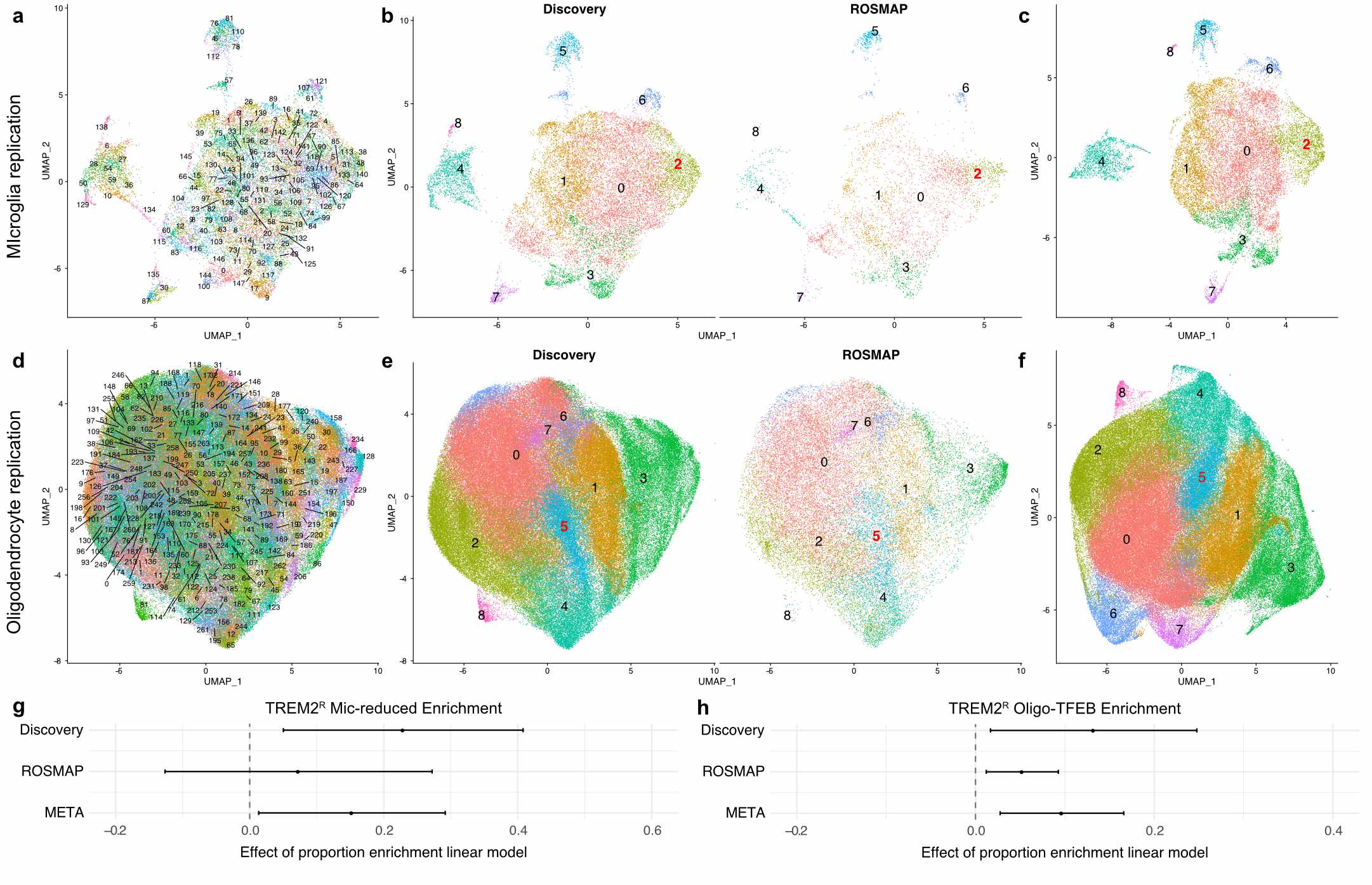
](https://wustl.box.com/s/los2yi5ni04j0omf8hts8a44hzyf9qvs)

Supplementary Figure 9 **TREM2 associated clusters replicated in ROSMAP cohort.**

**a,d)** UMAPs of discovery cohort (Knight ADRC and DIAN) and ROSMAP microglia (a) and oligodendrocytes (d) post-integration. A resolution of 15 was used to identify numerous clusters to facilitate mapping the discovery cohort cluster identities onto the ROSMAP nuclei. **b,e)** UMAPs of microglia (b) and oligodendrocytes (e) post cluster identity mapping, split by cohort. **c,f)** UMAPs of discovery cohort microglia (c) and oligodendrocytes (f) before ROSMAP integration. **g,h)** Forest plots of the Mic-reduced (cluster 2) in (g) and Oligo-TFEB (cluster 5) in (h) enrichment within TREM2 reduced activation variant carriers (TREM2^R^). The linear regression effect values (center) ± 1 standard error (bars) are shown for the independent discovery and ROSMAP cohorts, as well as the merged meta-analysis results. **g)** Discovery: 55 total samples (11 TREM2^R^, 44 OTH). ROSMAP: 28 total samples (9 TREM2^R^, 19 OTH). **h)** Discovery: 30 total samples (9 TREM2^R^, 21 OTH). ROSMAP: 21 total samples (10 TREM2^R^, 11 OTH). Source data are provided as a Source Data file.

[
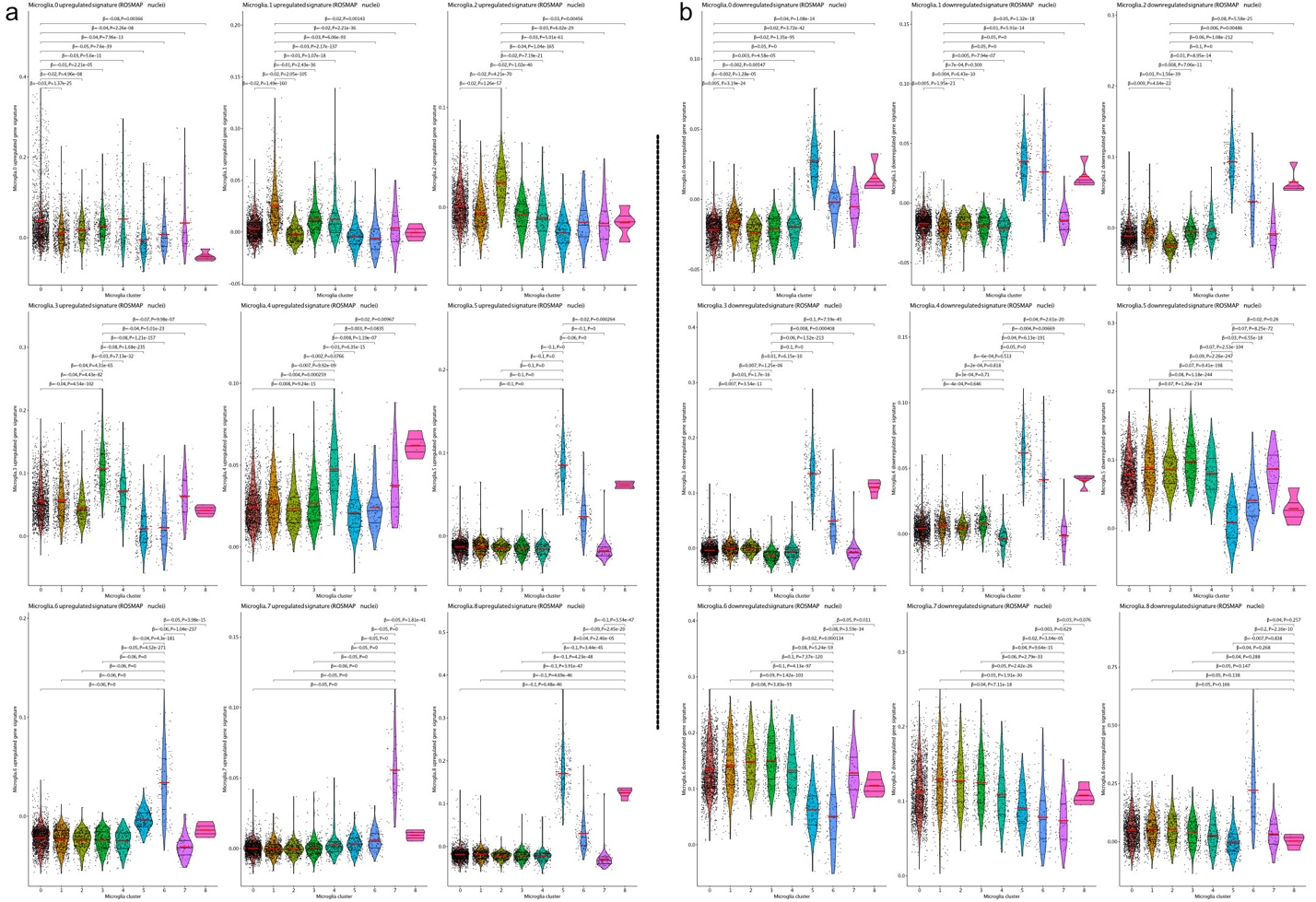
](https://wustl.box.com/s/oik0zzzzl9skwd2nmxblh8tqn7mi8cnu)

Supplementary Figure 10 ROSMAP microglia cell state signature scores.

Violin plots of the microglia cell state gene signature scores. The (a) upregulated and (b) downregulated genes from each microglia cell state in the discovery cohort were used to create scores for each nucleus in the ROSMAP cohort (see Methods). This method nicely validated the accuracy of label transfer from the discovery cohort onto the ROSMAP cohort. Source data are provided as a Source Data file.

[
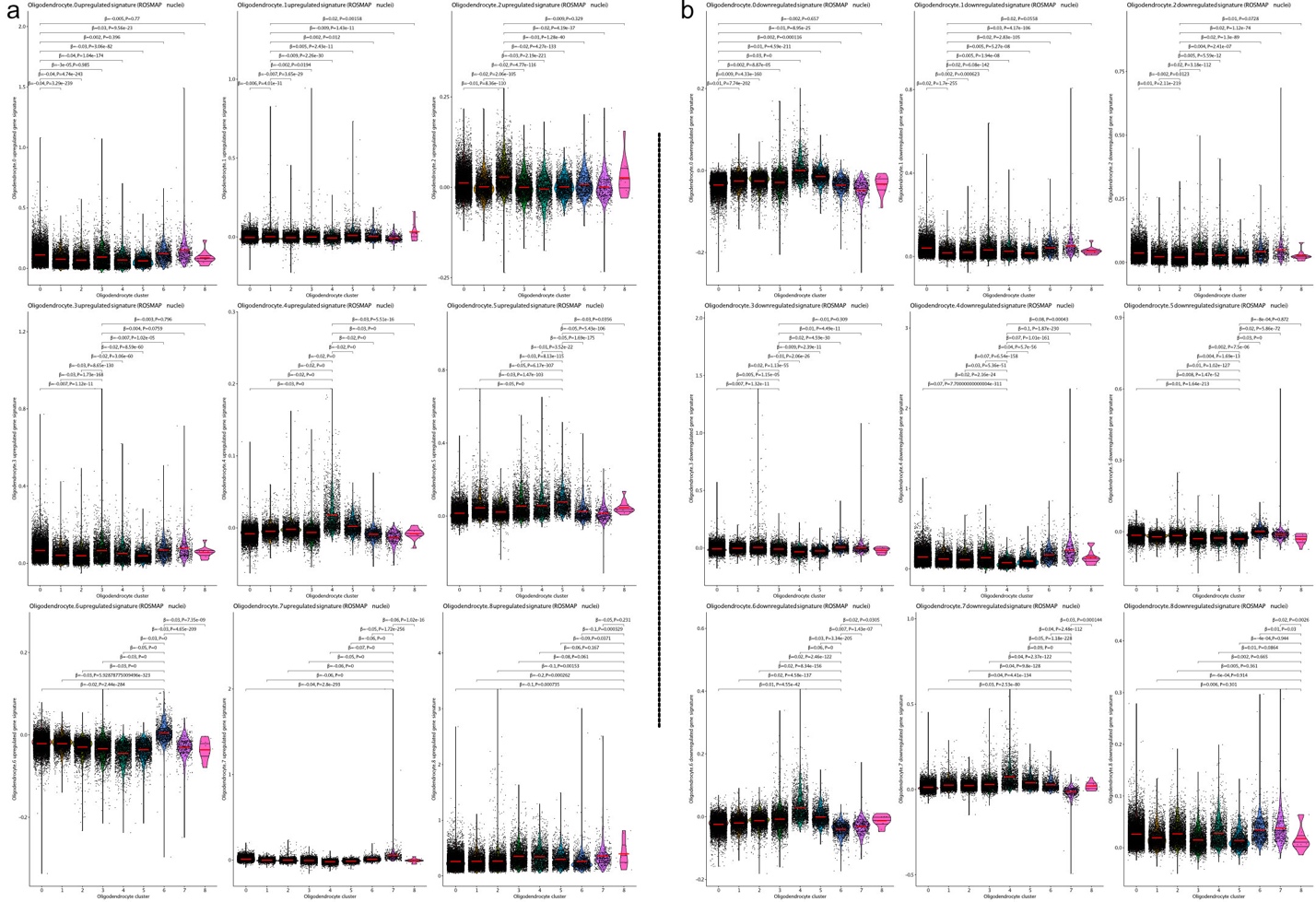
](https://wustl.box.com/s/70s1m48jpkbu421bhn7cs80f00bi7uv4)

Supplementary Figure 11 ROSMAP oligodendrocyte cell state signature scores.

Violin plots of the oligodendrocyte cell state gene signature scores. The (a) upregulated and (b) downregulated genes from each oligodendrocyte cell state in the discovery cohort were used to create scores for each nucleus in the ROSMAP cohort (see Methods). This method nicely validated the accuracy of label transfer from the discovery cohort onto the ROSMAP cohort. Source data are provided as a Source Data file.

[
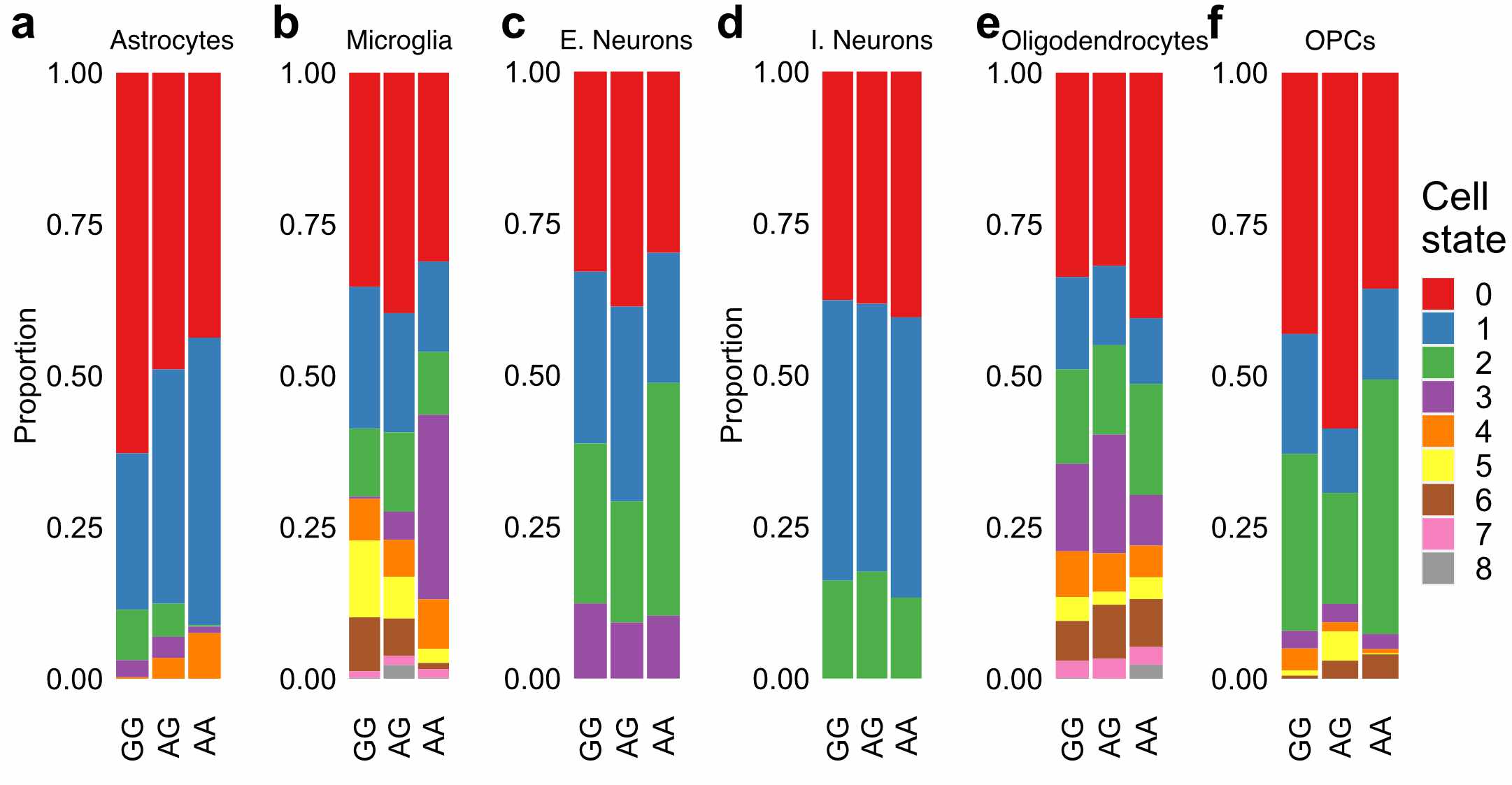
](https://wustl.box.com/s/v2cjbtwuj1rzs1b4fi7k95hhysio0vy0)

Supplementary Figure 12 MS4A cell state proportions by cell type.

Proportion plots showing the cell state proportions by MS4A (rs1582763) genotype for each cell type. The proportion was calculated for each sample (see Methods). For visualization, sample proportions are averaged by genotype. The 'G' allele is the wild type, and the 'A' allele is the alternate. **a)** Astrocytes. **b)** Microglia. **c)** Excitatory Neurons. **d)** Excitatory Neurons **e)** Oligodendrocytes. **f)** OPCs. Source data are provided as a Source Data file.

[
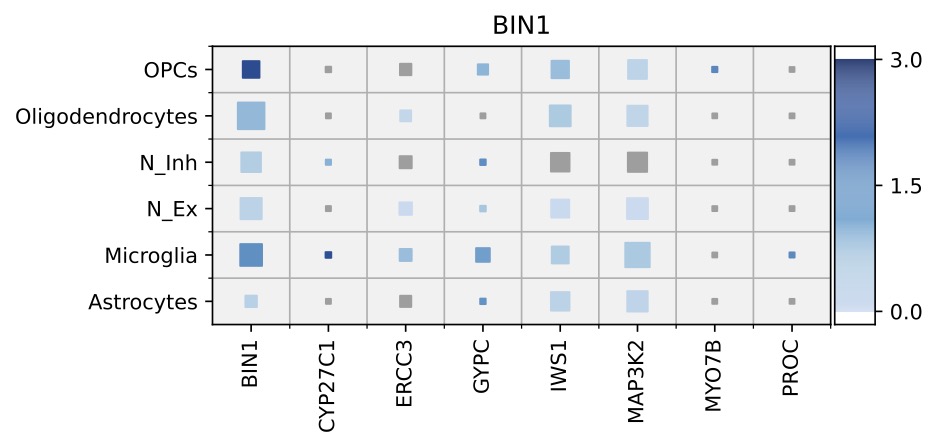
](https://wustl.box.com/s/jcagrrw27oven3l74g4354bwh51rvaf4)

Supplementary Figure 13 Expression Patterns of GWAS Loci.

These 46 plots are an extension of Figure 6. The idea behind these plots is that gene expression variability (differential expression) between cell states of the same cell type suggests an important function of the gene in that cell type. Some genes are only expressed in response to certain stimuli. These genes can show low average expression across the cell type even though they play a critical role in cell function. These plots highlight the cell types in which the GWAS loci genes could have functional importance. The color of the squares represents the max log2 fold change of the gene between cell states (subclusters) of that cell type. The log2 fold changes greater than three were set to three to preserve visual variability. Gray squares indicate no significant log2 fold change within that cell type. Square size represents the average log10 transformed gene expression. Click the figure or [here](https://wustl.box.com/s/wgi3cqcc76zgvqzjhpfw95ees0ekle3g) to see the remaining plots. Source data are provided as a Source Data file.

[
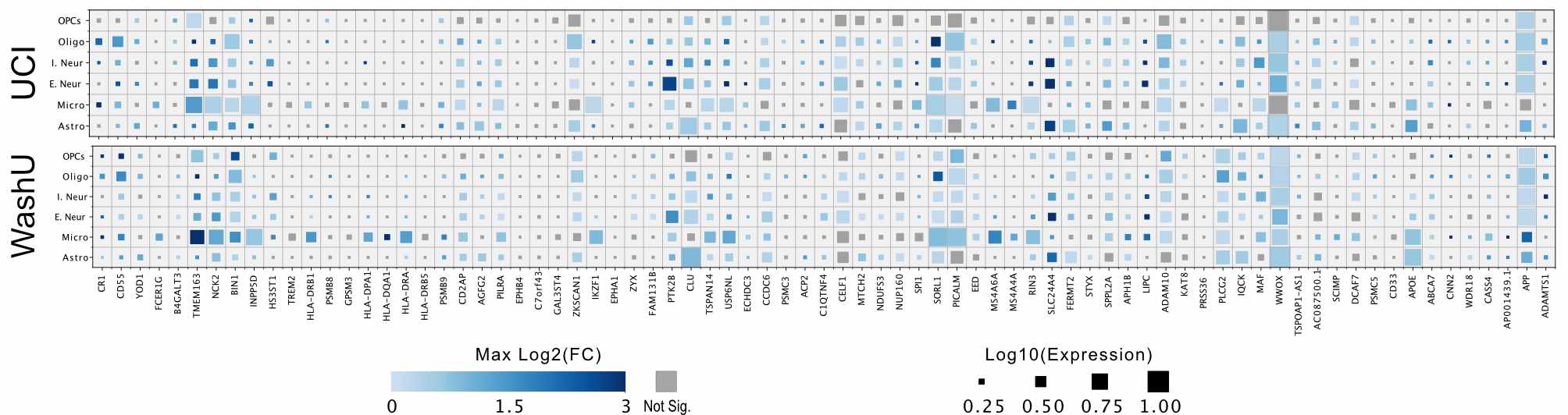
](https://wustl.box.com/s/qak8w6bjlgd6jl9w43nw322xfmjq60kv)

Supplementary Figure 14 Replication of prioritized GWAS gene expression patterns in UCI data.

This is a full side-by-side comparison of the prioritized GWAS gene expression patterns from the discovery (WashU) and replication (UCI) cohorts. As detailed in the Methods, we employed the data from UCI as processed by Morabito et al. The averaged expression and the max log2 fold changes within the cell types are remarkably similar and replicate in all the cell types except OPCs, possibly because of the lower number of OPC nuclei and clusters captured in the UCI data. A formal Fisher exact test was performed to quantify the concordance, and those results are included in Fig. 6. Source data are provided as a Source Data file.

[
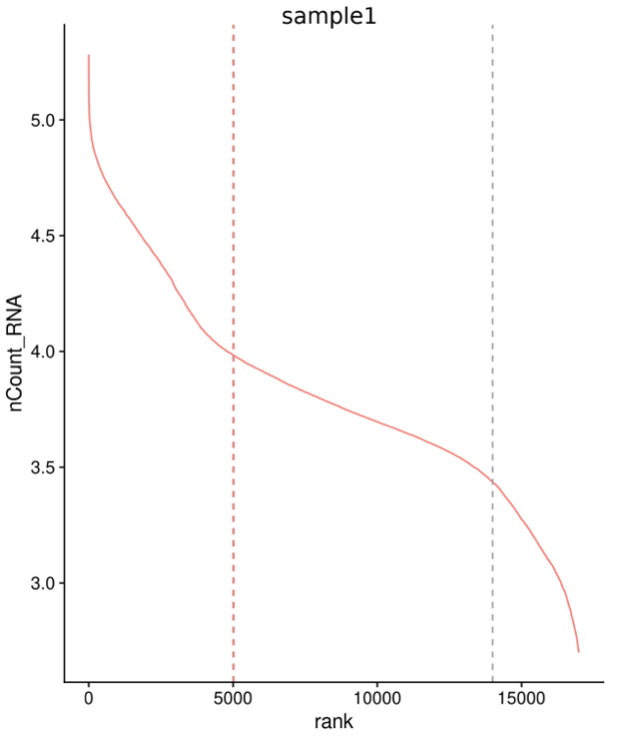
](https://wustl.box.com/s/zbepxhfxve2pqplvzgawma8sd9oiznph)

Supplementary Figure 15 Barcode inflection plots used in quality control filtering.

Barcode inflection plots order the nuclei by barcode counts for each sample. Gray lines indicated the upper and lower thresholds. The red line shows the calculated inflection point between the thresholds (sometimes it covers a gray threshold line). The tails on either end of the distribution frequently represent the doublets and empty cells that need to be removed before downstream analysis. Click the figure or [here](https://wustl.box.com/s/a16fieal36fggaojyql972e21vfkf9zr) to see the remaining plots.

[
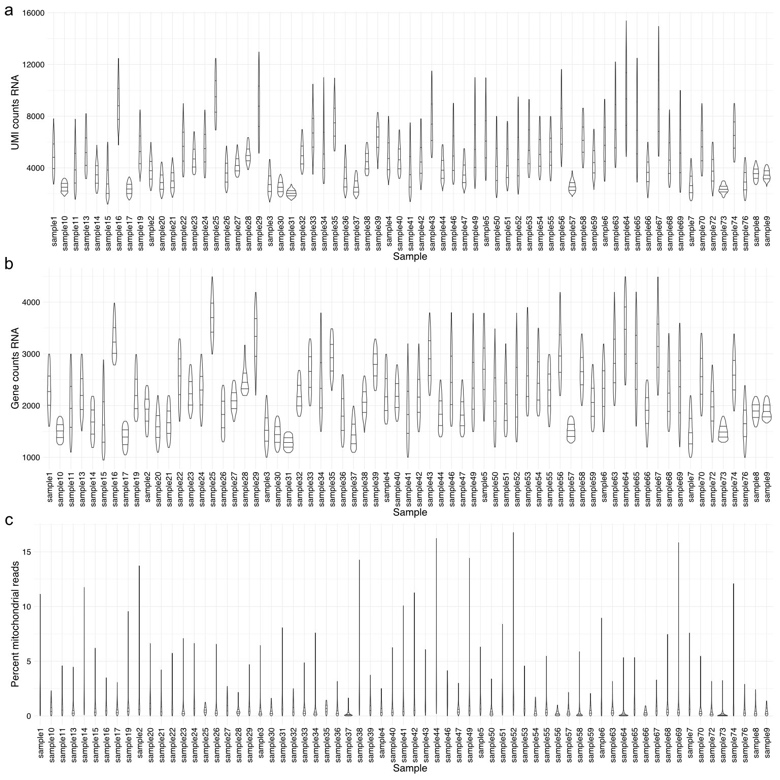
](https://wustl.box.com/s/nhf8zu1mxuzq77oot45jqwiv9d5juet1)

Supplementary Figure 16 QC metrics by sample.
Violin plots of UMI counts, gene counts and percent of mitochondrial reads for each sample.

[
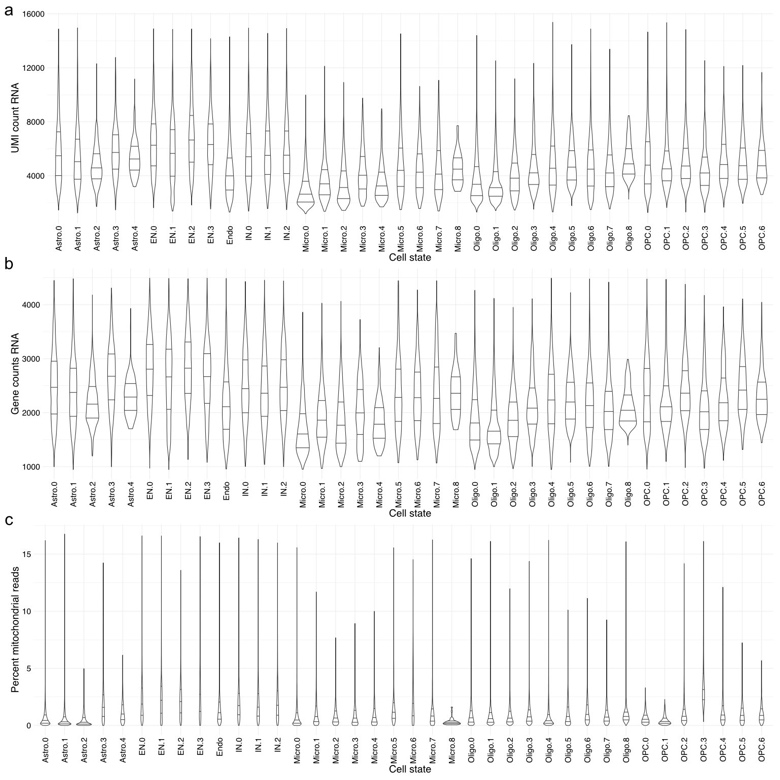
](https://wustl.box.com/s/45kly6rtkrokc7qghu3gux6afyt513ah)

Supplementary Figure 17 QC metrics by cell state.
Violin plots of UMI counts, gene counts and percent of mitochondrial reads for each cell state.

[
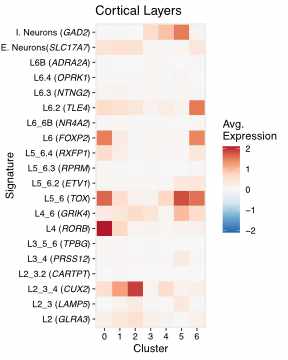
](https://wustl.box.com/s/0de5m5bt0mfr9dr4l4wi645ouwkh8978)

Supplementary Figure 18 Neuron cortical layers.
A heatmap of the average expression (accounting for sample) of neurons' inhibitory, excitatory, and cortical layer markers. These genes representing the category are in parentheses and were collected from Lake et al. Source data are provided as a Source Data file.

[
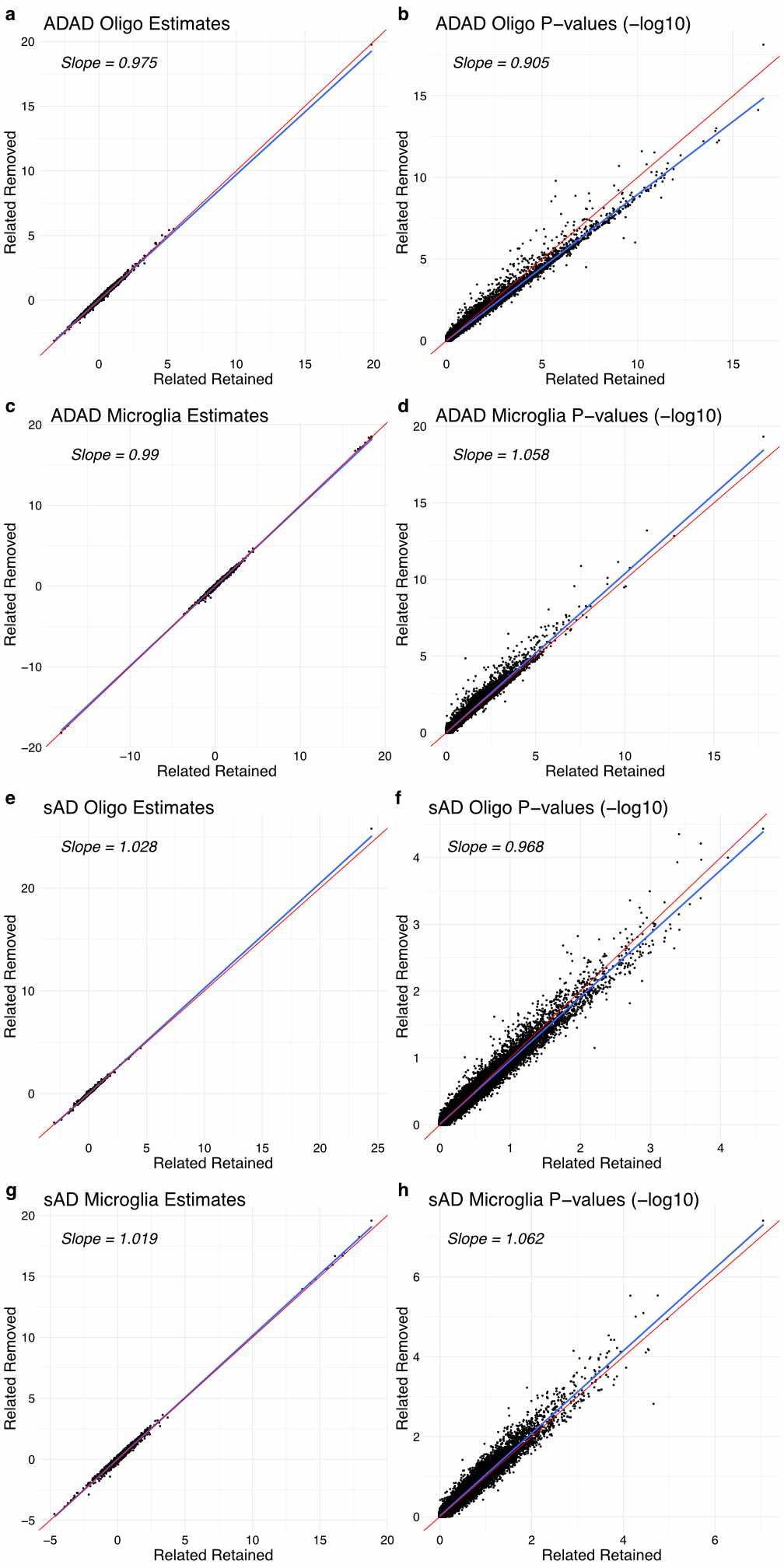
](https://wustl.box.com/s/o3dxbrkbh11o3y3g0eaqkzwwo2jo6s51)

Supplementary Figure 19 Comparing effects and p-values after removing genetically related individuals.

Scatterplots comparing the DEG effects (a,c,e,g) and p-values (b,d,f,h) within microglia and oligodendrocytes after removing a genetically related sample from the linear regression analysis. The blue line represents the line of best fit. The red line depicts a perfect 1:1 correlation. When the blue line is rotated clockwise compared to the red line (slope < 1), the effects/p-values are more extreme when retaining all the samples. In general, the estimates remained largely unaffected, but within oligodendrocytes, the p-values increased in significance when retaining both genetically related individuals, so this approach was used throughout the study. Source data are provided as a Source Data file.

[
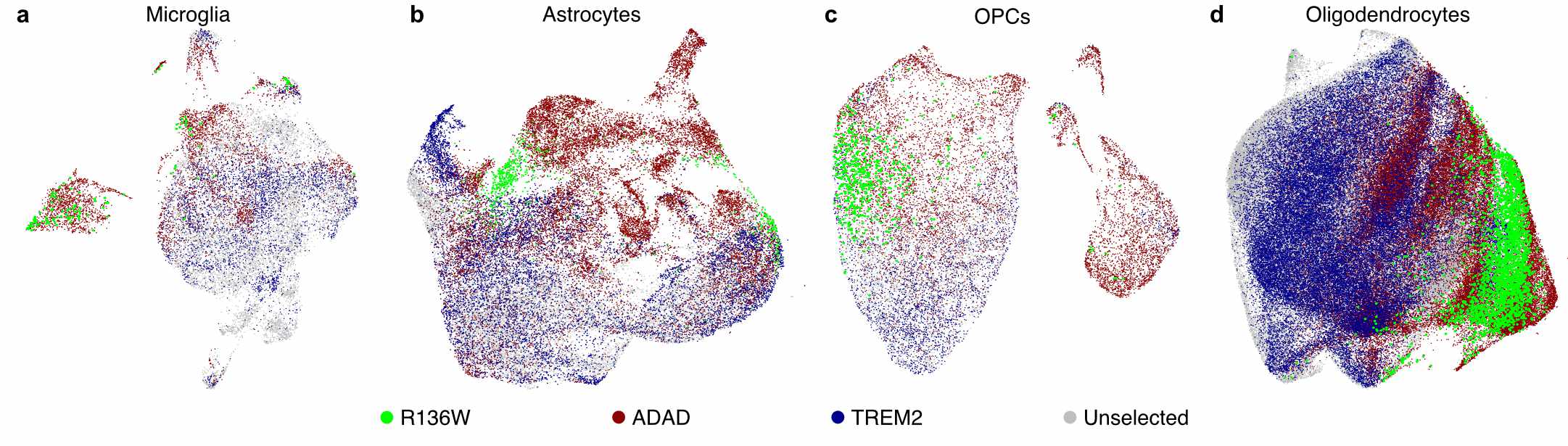
](https://wustl.box.com/s/xccorfge1z9qm2lk3vwahr2at60kpdku)

Supplementary Figure 20 TREM2 p.R136W clusters with ADAD.

TREM2 p.R136W carrier nuclei (green) cluster more closely with ADAD (red) than other TREM2 carriers (blue) in Microglia, Astrocytes, OPCs, and Oligodendrocytes (from left to right).

[
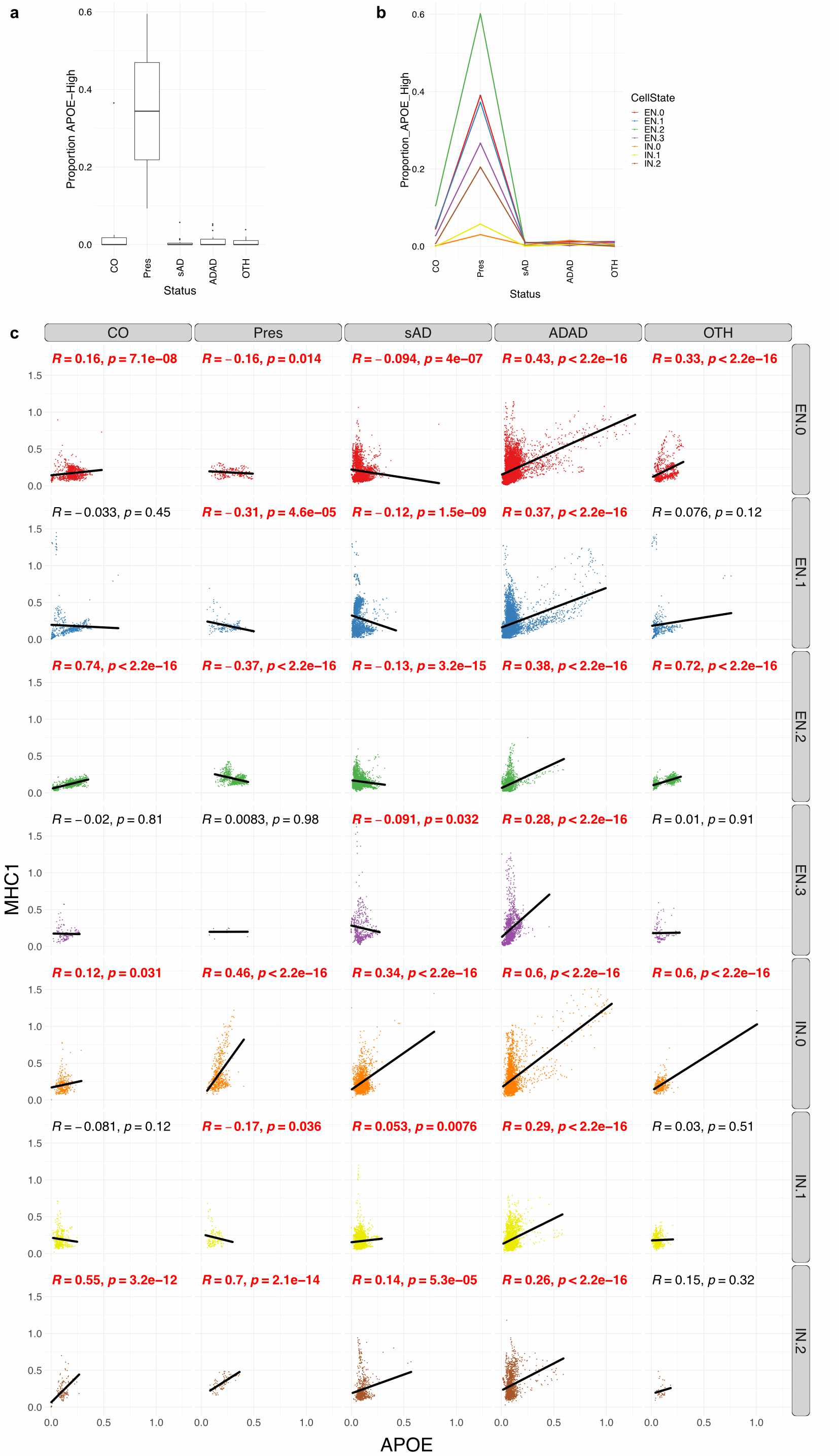
](https://wustl.box.com/s/5lytpuxezs896uwtlyz8c1412eaplegw)

Supplementary Figure 21 Neuronal APOE and MHC1 expression.

a) Boxplot showing the proportion of APOE-high neurons in each sample by status. Center line, median; box limits, upper and lower quartiles; whiskers, non-outlier max and min; points, outliers (1.5*IQR). CO = neuropath free (n=9 samples); Pres = presymptomatic (n=2); sAD = sporadic AD (n=28); ADAD = autosomal dominant AD (n=15); OTH = non-AD neurodegenerative (n=7). b) The average proportion of APOE-high neurons in each status is split by cell state. EN = excitatory neurons, IN = inhibitory neurons. c) Scatter plots and regression lines depicting the coexpression between APOE and MHC-I in neurons after MAGIC imputation. Bold red text highlights the significant correlations as calculated by the Spearman correlation test. Source data are provided as a Source Data file.

# Dominantly Inherited Alzheimer Network (DIAN) Author List

Dave Cash^16,17^, Nick C. Fox^16,17^, Jonathan Voglein^18,19^, Richard J Perrin^2,6,8,9^, Carlos Cruchaga^1,3^, Victoria Fernanadez^1,3^, Johannes Levin^19,20^, Anna Hofmann^19,21^, Mathias Jucker^19,21^, Christoph Laske^19,21^, Oliver Preische^19,21^, William S. Brooks^22,23^, Peter R. Schofield^22,23^, Raquel Sanchez-Valle^24^, Takeshi Ikeuchi^25^, Kensaku Kasuga^25^, Courtney Bodge^26^, William Menard^26^, Meghan C. Riddle^26^, Mustafa Surti^26^, Ricardo F. Allegri^27^, Patricio Chrem Mendez^27^, Bianca T. Esposito^28^, Alan E. Renton^29^, Ezequiel Surace^30^, John Ringman^31^, Snezana Ikonomovic^32^, Anne M. Fagan^9^, Elizabeth Herries^9^, Gina Jerome^9^, Eric McDade^1^, Charlene Supnet-Bell^9^, Erin E. Franklin^6^, Celeste M. Karch^1,2,3^, Jacob Marsh^33^, Brian A. Gordon^34^, Jinbin Xu^34^, Neelesh K. Nadkarni^35^, Sarah B. Berman^36^, Jason Hassenstab^37^, VJ Sanchez-Gonzalez^38^, Maribel Orozco-Barajas^39^, Ralph Martins^40^, Susanne Graber-Sultan^19^, Elke Kuder-Buletta^19^, Erik C.B. Johnson^41^, Allan I. Levey^41^, Nicholas T. Seyfried^41^, David Aguillon^42^, Diana Alzate^42^, Ana Baena^42^, Yudy Leon^42^, Natalia Londono^42^, Francisco Lopera^42^, Sonia Moreno^42^, Laura Ramirez^42^, Claudia Ramos^42^, Leonel Tadao Takada^43^, Martin Farlow^13^, Ana Luisa Sosa Ortiz^45^, Michio Senda^46^, Jae-Hong Lee^47^, Jee Hoon Roh^47^, Jasmeer P. Chhatwal^48^, Gregory S. Day^49^, Neill R. Graff-Radford^49^, Pedro Rosa-Neto^50^, Jacob A Bechara^22^, Hiroshi Mori^51^, Alison M. Goate^12^, James M. Noble^52^, Kenji Ishii^53^, Yoshiki Niimi^54^, Randall J Bateman^2,8,9^, Tammie Benzinger^55^, Ryan Bui^55^, Madison Candela^55^, Allison Chen^55^, Charles Chen^55^, Laura Courtney^55^, Alisha Daniels^55^, Emily Deng^55^, Shaney Flores^55^, Nelly Friedrichsen^55^, Brian Gordon^55^, Emily Gremminger^55^, Nancy Hantler^55^, Diana Hobbs^55^, Russ Hornbeck ^55^, Steve Jarman^55^, Sarah Keefe^55^, Deborah Koudelis^55^, Yan Li^55^, Jorge Llibre-Guerra^55^, Ruijin Lu^55^, Yinjiao Ma ^55^, Parinaz Massoumzadeh^55^, Austin McCullough^55^, Nicole McKay^55^, Sheetal Mishall^55^, Joyce Nicklaus^55^, Christine Pulizos^55^, Edita Sabaredzovic^55^, Jalen Scott^55^, Hunter Smith^55^, Qing Wang^55^, Peter Wang^55^, Chengjie Xiong^8,11^, Xiong Xu^55^

Affiliations:

1. Department of Psychiatry, Washington University School of Medicine in St. Louis, St. Louis, MO, USA
2. Hope Center for Neurological Disorders, Washington University School of Medicine in St. Louis, St. Louis, MO, USA
3. NeuroGenomics and Informatics, Department of Psychiatry, Washington University School of Medicine in St. Louis, St. Louis, MO, USA
4. Merck & Co., Inc., Boston, MA, USA
5. Baylor College of Medicine, Houston, TX, USA
6. Department of Pathology and Immunology, Washington University School of Medicine in St. Louis, St. Louis, MO, USA
7. Center for Brain Immunology and Glia (BIG), Washington University School of Medicine in St. Louis, St. Louis, MO, USA
8. Knight Alzheimer Disease Research Center, Washington University School of Medicine in St. Louis, St. Louis, MO, USA
9. Department of Neurology, Washington University School of Medicine in St. Louis, St. Louis, MO, USA
10. Dominantly Inherited Alzheimer Network (DIAN)
11. Division of Biostatistics, Washington University School of Medicine in St. Louis, St. Louis, MO, USA
12. Ronald M. Loeb Center for Alzheimer’s Disease, Department of Genetics and Genomic Sciences, Icahn School of Medicine at Mount Sinai, New York, NY, USA
13. Department of Neurology, Indiana University School of Medicine, Indianapolis, IN, USA
14. School of Medical Sciences and Charles Perkins Centre, Faculty of Medicine and Health, The University of Sydney, Sydney, NSW, Australia
15. Department of Neurology, Beth Israel Deaconess Medical Center, Harvard Medical School, Boston, MA, USA
16. Dementia Research Centre, UCL Queen Square Institute of Neurology, London, UK
17. UK Dementia Research Institute at UCL, London, UK
18. Departmant of Neurology, Ludwig-Maximilians-Universitat Munchen, Munich, Germany
19. German Center for Neurodegenerative Diseases (DZNE), Munich, Germany
20. Department of Neurology, Ludwig-Maximilians-Universität München, Munich, Germany 3) Munich Cluster for Systems Neurology (SyNergy), Munich, Germany
21. Hertie-Institute for Clinical Brain Research, Tübingen, Germany
22. Neuroscience Research Australia, Sydney, Australia
23. School of Medical Sciences, University of New South Wales, Sydney, Australia
24. Alzheimer's disease and other cognitive disorders Unit, Neurology Service, Hospital Clinic de Barcelona, Barcelona, Spain
25. Brain Research Institute, Niigata University, Niigata, Japan
26. Butler Hospital, Warren Alpert School of Medicine at Brown University, Providence, RI, USA
27. Department of Cognitive Neurology, Institute for Neurological Research Fleni, Buenos Aires, Argentina
28. Department of Genetics and Genomic Sciences and Ronald M. Loeb Center for Alzheimer's Disease, Icahn School of Medicine at Mount Sinai, New Youk, NY, USA
29. Department of Genetics and Genomic Sciences, Nash Family Department of Neuroscience, and Ronald M. Loeb Center for Alzheimer's Disease, Icahn School of Medicine at Mount Sinai, New Youk, NY, USA
30. Department of Molecular Biology and Neuropathology, Institute for Neurological Research Fleni, Buenos Aires, Argentina
31. Department of Neurology, Keck School of Medicine of USC, Universty of Southern California
32. Department of Neurology, University of Pittsburgh, Pittsburgh, PA, USA
33. Department of Psychiatry, Washington University School of Medicine in St. Louis
34. Department of Radiology, Washington University School of Medicine in St. Louis, St. Louis, MO, USA
35. Departments of Medicine (Geriatric Medicine) and Neurology, University of Pittsburgh, Pittsburgh, PA, USA
36. Departments of Neurology and Clinical & Translational Science, University of Pittsburgh, Pittsburgh, PA, USA
37. Departments of Neurology and Psychological & Brain Sciences, Washington University School of Medicine in St. Louis, St. Louis, MO, USA
38. Doctorado en Biociencias & Departamento de Clinicas, Centro Universitario de Los Altos, UDG
39. Doctorado en Biociencias & Departamento de Salud, Centro Universitario de Los Altos, UDG
40. Edith Cowan University, Joondalup, Australia
41. Goizueta Alzheimer's Disease Research Center, Emory University, Atlanta, GA, USA
42. Grupo de Neurociencias de Antioquia (GNA), Universidad de Antioquia, Medellin, Colombia
43. Hospital das Clinicas, University of Sao Paulo School of Medicine, Sao Paulo, Brazil
44. Indiana University, Bloomington, IN, USA
45. Instituto Nacional de Neurología y Neurocirugía Manuel Velasco Suárez: Mexico City, Mexico City, MX
46. Kobe City Medical Center General Hospital, Kobe, Japan
47. Korea University College of Medicine, Seoul, South Korea
48. Massachusetts General Hospital, Brigham and Women's Hospital, Harvard Medical School, Boston, MA, USA
49. Mayo Clinic , Department of Neurology, Jacksonville, Flordia, USA
50. McGill University, Montreal, Quebec, Canada
51. Osaka Metropolitan University, Osaka, Japan
52. Taub Institute for Research on Alzheimer's Disease and the Aging Brain, G.H. Sergievsky Center, Department of Neurology, Columbia University Irving Medical Center, New York City, NY, USA
53. Tokyo Metropolitan Institute of Gerontology, Tokyo, Japan
54. Unit for Early and Exploratory Clinical Development, The University of Tokyo Hospital, Tokyo, Japan
