# Supplementary material for: Single-nucleus RNA-sequencing of autosomal dominant Alzheimer disease and risk variant carriers
Source: Nat Commun. 2023 Apr 21;14:2314. doi: 10.1038/s41467-023-37437-5 (PMC10121712; doi:10.1038/s41467-023-37437-5)
Supplement: Supplementary file 3 — Description of Additional Supplementary Files [file 41467_2023_37437_MOESM3_ESM.pdf]

Description of Additional Supplementary Files

File Name: Supplementary Dataset 1
Description: Sample genotype statuses

File Name: Supplementary Dataset 2
Description: Cell type details

File Name: Supplementary Dataset 3
Description: Cell type marker genes

File Name: Supplementary Dataset 4
Description: Cluster distributions

File Name: Supplementary Dataset 5
Description: Batch and sample entropy calculations

File Name: Supplementary Dataset 6
Description: Cluster proportion results

File Name: Supplementary Dataset 7
Description: Number of upregulated genes per subcluster

File Name: Supplementary Dataset 8
Description: DEGs by cell state

File Name: Supplementary Dataset 9
Description: Sample and nuclei counts for linear regression analyses

File Name: Supplementary Dataset 10
Description: DEGs by AD status

File Name: Supplementary Dataset 11
Description: Number of DEGs by AD status

File Name: Supplementary Dataset 12
Description: Pathway results for expression pattern modules

File Name: Supplementary Dataset 13
Description: Overlapping DEGs between cell types within genetic groups

File Name: Supplementary Dataset 14
Description: Pathway results between cell types within genetic groups

File Name: Supplementary Dataset 15
Description: Overlapping DEGs between genetic groups within cell types

File Name: Supplementary Dataset 16
Description: Pathway results between genetic groups within cell types

File Name: Supplementary Dataset 17
Description: Cell state pathway results

File Name: Supplementary Dataset 18
Description: Microglia hypergeometric

File Name: Supplementary Dataset 19
Description: Replication of pySCENIC regulons in the ROSMAP cohort

File Name: Supplementary Dataset 20
Description: Mic-activated (Mic.1) v Mic-proinflammatory (Mic.3) DEGs

File Name: Supplementary Dataset 21
Description: APOE pathway results

File Name: Supplementary Dataset 22
Description: GWAS genes

File Name: Supplementary Dataset 23
Description: UCI cell states used in replicating the GWAS loci prioritization

File Name: Supplementary Dataset 24
Description: RIN scores from bulk RNA for each sample

File Name: Supplementary Dataset 25
Description: Threshold filters for barcode inflection plots

File Name: Supplementary Dataset 26
Description: Genetically related samples

File Name: Supplementary Dataset 27
Description: Expression pattern modules by cell type

File Name: Supplementary Dataset 28
Description: Number of genes used for cell state signature score calculation

File Name: Supplementary Dataset 29
Description: APOE-high neuron DEGs

File Name: Supplementary Dataset 30
Description: APOE-high pathway results
